# The paradox of falling job satisfaction with rising job stickiness in the German nursing workforce between 1990 and 2013

**DOI:** 10.1186/s12960-017-0228-x

**Published:** 2017-08-29

**Authors:** Mohamad Alameddine, Jan Michael Bauer, Martin Richter, Alfonso Sousa-Poza

**Affiliations:** 1 College of Medicine, Directorate of Strategy and Institutional Excellence, Mohammed Bin Rashid University of Medicine and Health Sciences, P.O. Box 505055, Dubai, United Arab Emirates; 2 Department of Health Management and Policy, Faculty of Health Sciences, American University of Beirut, Beirut, Lebanon; 3 Department of Management, Society and Communication, Copenhagen Business School, Porcelænshaven 18, 2000 Frederiksberg, Denmark; 4 Institute for Healthcare & Public Management (530A), University of Hohenheim, Fruwirthstr. 48, 70599 Stuttgart, Germany

**Keywords:** Nursing, Job satisfaction, Germany, Job stickiness

## Abstract

**Background:**

Literature reports a direct relation between nurses’ job satisfaction and their job retention (stickiness). The proper planning and management of the nursing labor market necessitates the understanding of job satisfaction and retention trends. The objectives of the study are to identify trends in, and the interrelation between, the job satisfaction and job stickiness of German nurses in the 1990–2013 period using a flexible specification for job satisfaction that includes different time periods and to also identify the main determinants of nurse job stickiness in Germany and test whether these determinants have changed over the last two decades.

**Methods:**

The development of job stickiness in Germany is depicted by a subset of data from the German Socio-Economic Panel (1990–2013), with each survey respondent assigned a unique identifier used to calculate the year-to-year transition probability of remaining in the current position. The changing association between job satisfaction and job stickiness is measured using job satisfaction data and multivariate regressions assessing whether certain job stickiness determinants have changed over the study period.

**Results:**

Between 1990 and 2013, the job stickiness of German nurses increased from 83 to 91%, while their job satisfaction underwent a steady and gradual decline, dropping by 7.5%. We attribute this paradoxical result to the changing association between job satisfaction and job stickiness; that is, for a given level of job (dis)satisfaction, nurses show a higher stickiness rate in more recent years than in the past, which might be partially explained by the rise in part-time employment during this period. The main determinants of stickiness, whose importance has not changed in the past two decades, are wages, tenure, personal health, and household structure.

**Conclusions:**

The paradoxical relation between job satisfaction and job stickiness in the German nursing context could be explained by historical downsizing trends in hospitals, an East-West German nurse compensation gap, and an increase in the proportion of nurses employed on a part-time basis. A clearer analysis of each of these trends is thus essential for the development of evidence-based policies that enhance the job satisfaction and efficiency of the German nursing workforce.

## Background

Nursing shortages is a growing global concern because of its major implications for patient outcomes and the quality of care provided [1, 2]. A major factor underlying these shortages is turnover, the rate at which a healthcare organization gains or loses nurses over a certain period of time [3]. The design, implementation, and evaluation of optimal strategies to enhance the retention of the nursing workforce are essential to decrease their high levels of turnover [3]. In fact, the annual turnover rate in the nursing workforce is higher than the turnover rates in any other profession, reaching between 9 and 15% in Germany, Italy, Finland, France, and the UK [4]. Among nurses under the age of 30 in Germany, Canada, the USA, England, and Scotland, 27–54% reported plans to leave their positions within the coming 12 months [5]. A more recent study similarly found that 17, 30, 39, and 23% of nurses intended to leave their current job in the next year in Germany, Scotland, England, and the USA, respectively [5]. Understanding the underlying causes of nurse turnover is critical because of the resulting costs and consequences, which are borne by both organizations and patients [6–9]. Healthcare organizations need to invest heavily in recruiting and training new staff while attempting to balance the work overload on the remaining nurses [10, 11]. This in turn may result in higher burnout and dissatisfaction for staff and poor health outcomes for patients [2, 12, 13].

Although turnover can be captured in several ways, perhaps the most common method is to analyze turnover intentions [14], which often serve as a precursor to actual turnovers [15]. However, turnover intentions, although conveniently measured with cross-sectional data, are a mere proxy of actual turnovers. Another common alternative for cross-sectional data is reported tenure, but this variable tends to capture only *elapsed* tenure, which is right-censored. A frequently used measure of job stability in the labor economics literature is 1-year job separations [16], which have the advantage of directly measuring separation; however, application in the nursing market is limited by insufficient panel data to identify actual turnover. In this study, we use the inverse of 1-year separations, commonly referred to as “stickiness,” which we define as the retention probability, i.e., that the same employee working in a specific sector/subsector of employment in year *t* will remain working in the same sector/subsector in year *t* + 1 [17]. This measure has been previously used to proxy the relative attractiveness of various healthcare sectors of nursing employment over time [17, 18].

The relationship between job satisfaction and retention is very well established [11]. A study on turnover in the health sector indicates that although several factors can influence turnover, job dissatisfaction remains its most consistent predictor [19]. The link between job satisfaction and turnover *intentions* has also been well-documented [20]. Similarly, examined from a retention perspective, job satisfaction is the foremost indicator of the likelihood that an employee will remain in his/her position [21]. Thus, healthcare leaders aim to develop and maintain empowering work environments to enhance employees’ job satisfaction as a strategy to improve their HR retention [22, 23].

In this paper, we analyze the job stickiness of nurses within the German health market and test for any association between job stickiness and job satisfaction levels. Of particular interest is the temporal dimension; that is, the evolution of job satisfaction and stickiness across time. Our main research questions are as follows: (i) How has job satisfaction and stickiness evolved in Germany’s nursing market since 1990? (ii) What relation exists between job satisfaction and stickiness and has this relation changed over time? (iii) Does this relation differ across (health-care and service) sectors and job characteristics? (iv) Have the main determinants of job satisfaction changed over time?

The significance of this study is three-pronged. First, to the best of our knowledge, this study is the first to use panel data to document trends in nursing turnover over an extended period of time (nearly a quarter of a century). Second, it is also the first study in Germany that analyzes job turnover in the nursing labor force using *actual* turnover instead of turnover intentions. Such analyses are rare with noticeable exceptions [24]. Lastly, our study serves as a primer for how to uncover a more in-depth relation between job satisfaction and labor turnover, not only with regard to specification but also changing relations across time.

### Institutional framework

#### Nurses in Germany

Nursing education programs in Germany comprise 3 years of vocational training offered and run by accredited hospitals, and the curriculum is determined by state guidelines and county law. During the training period, nursing students are paid by the provider and are part of this hospital’s nursing workforce. This on-the-job training promotes job-related skills and enhances the nurse-patient communication experience. Each vocational training program consists of at least 4600 h, with a minimum of 2300 h dedicated to practical education and no less than one third dedicated to the nursing program’s theoretical component. The proportional distribution of regular nurses and nursing assistants depends on the type of care institution with the former constituting the majority of nurses in hospitals and the later comprising the majority of nurses in elder care facilities [25].

A recent study revealed a large drop in the satisfaction levels of nurses in Germany [26]. Between 1991 and 2014, the cases per year for each full-time equivalent (FTE) nurse increased by 34.4%, leading to an 18% decline in the perceived adequacy of nurse staffing between 1999 and 2009 [27]. The last 25 years also witnessed a changing employment pattern for German nurses, with a 51.0% increase (East + 108.7%, West + 44.6%) in the share of part-time employment among non-physician hospital staff between 1991 and 2003, and a 21.0% (East + 52.4%, West + 17.4%) increase between 2003 and 2014. This could be attributed to personal and family reasons or decreased availability of full-time employment opportunities [25].

## Methods

### Data and sample

The German Socio-Economic Panel (GSOEP) has been administered nationally since 1984. It is one of the longest running longitudinal, national, population-based household surveys in the world. GSOEP employs a regionally clustered, multistage sampling procedure to collect data from adults within selected households. It has been refreshed five times over the last 30 years using survey weights to replace dropouts and restore its representativeness of the German population. GSOEP information is collected via an annual survey in which individuals indicate their current situation or reflect on certain life events that have occurred since the last interview. The data aims to provide a consistent representation of the German population and administrators closely monitor data quality. The GSOEP has been reviewed by the British Economic and Social Research Council and the data is collected with an ISO certificate since 1995. The number of surveyed individuals has grown gradually over the years, from 12 000 to around 20 000 in 2012.

This study utilizes data from 24 waves (1990 to 2013) of GSOEP. The dataset provides a rich set of work-related variables, including living conditions, economic well-being, household earnings, employment status, occupational sector, work hours, job satisfaction, and working conditions. The main analysis in this study uses data on a subset of German nurses from 1990 (German unification) to 2013 (the latest available wave). Our primary sample of interest is nurses who reported being active in the labor force at least once during the study period. Nurses are defined according to the German StaBuA92 classification code 8530-8539 equivalent to the ISCO88 code 3231 “Nursing associate professionals”. Our final sample includes 5114 person-year observations for 997 individual nurses (see Appendix 1 for the sample sizes in each wave). Because we are interested in identifying changes across these 24 years, most analyses are performed separately for two almost equal time periods, 1990–2002 and 2003–2013, into which we partition our sample. As shown below, neither the trends in job satisfaction nor those in job turnover show any marked structural shifts, so partitioning the 24 years of data in this manner is a feasible approach to creating two equally sized samples.

### Stickiness

In this study, job stickiness is defined based on three criteria: (1) the individual reports being employed as a nurse in both the current year (*t*) and the next survey year (*t* + 1), (2) the individual retains at least the current level of employment with no reduction in working hours, and (3) the individual does not change the current employer or job position with this employer. Thus, we use a refined measure of stickiness which encompasses not only occupational but also job turnover.

#### Job satisfaction

GSOEP measures job satisfaction on a 10-point ordinal scale from 0 = totally unhappy to 10 = totally happy based on the following item: “How satisfied are you with your job?”. The use of single-item job satisfaction measures has shown to provide valid and reliable results in the literature [28, 29]. There is also a large body of literature in labor economics which shows that such single-item measures also predict future behavior (e.g., job changes) very well [30].

#### Job satisfaction-stickiness relation

To measure the association between job satisfaction level and stickiness level, we estimate the following multivariate linear probability model using ordinary least squares (OLS):

1 $$ \mathrm{stick}{\mathrm{y}}_{s,i,t+1}={\beta}_0+{\beta}_1f\left(\mathrm{job}\ \mathrm{satisfactio}{\mathrm{n}}_{s,i,t}\right)+{\mathrm{state}}_s+{\mathrm{y}\mathrm{ear}}_t+{\epsilon}_{s,i,t} $$

where the dependent variable sticky_*s* , *i* , *t* + 1_ is a dummy variable equal to 1 if a nurse retains the current position and working hours in the next survey year; and 0 otherwise. To determine the probability that the nurse will remain in the same position in the following year, we regress the stickiness variable on a function of current nurse job satisfaction, which is made flexible in the regression through the use of a linear, squared, and cubic specification of job satisfaction. We also include dummies for federal state_*s*_ and survey year_*t*_ to control for overall trends and idiosyncratic shocks during the survey period. *ϵ*_*s* , *i* , *t*_ indicates the heteroskedastic robust standard errors, which are clustered at the individual level. We, thereby, account for the repeated observation of the same individuals, which are not independent from each other. We also include survey weights to restore the representatives of data distorted by survey administrator oversampling of certain subpopulations.

### Further determinants of stickiness

To analyze other potential determinants of stickiness, we incorporate a large set of explanatory variables into model (1), again using OLS to estimate a function of the following form:

2 $$ \mathrm{stick}{\mathrm{y}}_{s,i,t+1}={\beta}_0+{\beta}_1{X}_{s,i,t}+{\mathrm{state}}_s+{\mathrm{y}\mathrm{ear}}_t+{\epsilon}_{s,i,t} $$

We now control for a vector of socioeconomic covariates to account for other determinants *X*_*s* , *i* , *t*_ that might affect the probability of a job change. Because job satisfaction is a catch-all variable, we omit it from this specification. Rather, *X*_*s* , *i* , *t*_ covers gender, age, years of education, marital status, current level of employment, number of children in the household, migrant background, annual number of doctor visits years in the company, actual working hours, and net household income. Although our choice of explanatory variables is primarily driven by the available data in the GSOEP, all these covariates have been shown to be related to turnover. Women, combined with marital status, the level of employment, as well as the number of children in the household, are all associated with kinship responsibilities, which tend to weaken the attachment to the work environment [11]. Several studies have shown that age has an inverse relation with turnover [31]. Possible reasons include that older nurses have more firm-specific knowledge and tend to have higher levels of job satisfaction. Higher levels of education often go hand-in-hand with higher turnover rates as they are more likely and able to advance their careers by job changes [32, 33]. High education is also often associated with better labor-market alternatives [34]. In much of the economic literature, an inverse relationship between the wage rate or income and the probability of a job change is assumed [35, 36]. The reason hereto is that the higher the actual wage rate is, the lower the probability will be of finding an employer offering a higher wage rate [37]. Evidence on the effect of income on turnovers in the nursing market is, however, inconclusive [11]. Migrants have also been shown to have higher fluctuation rates as they explore the labor market by trial-and-error [38]. Less healthy individuals are more likely to leave their jobs [39]. Similar to regression (1), we add federal state and survey year dummies.

## Results

**Fig. 1:**
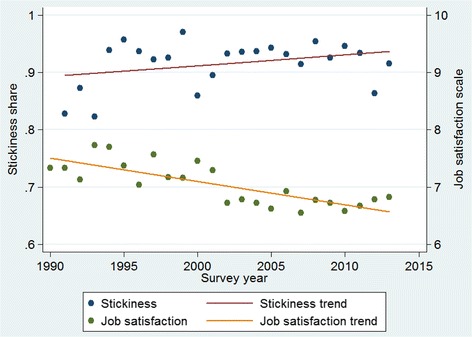
Trends for job stickiness and job satisfaction

Relevant descriptive statistics for the two time periods (1990–2002 and 2003–2013) are reported in Table 1, which reveals three clear trends: a statistically significant reduction in the proportion of female nurses, an increase in part-time employment, and a decline in job satisfaction. Average stickiness in the two periods, on the other hand, is very similar (91.2 and 92.9%, respectively), indicating that turnover rates have little changed in these 24 years. Figure 1 then graphs the trends in job satisfaction and stickiness over the study period, showing an approximate 1 point (on a 10-point scale) decrease in average job satisfaction between 1990 and 2013. Conversely, job stickiness shows a slight upward trend from approximately 0.9 in 1990 to 0.92 in 2013. As Fig. 1 indicates, there is some fluctuation in job stickiness, which to some extent can be explained by business-cycle developments (the correlation between stickiness and the unemployment rate is equal to 0.40).

**Table 1:** Descriptive statistics

	1990–2002	2003–2013
Age (years)	35.7 (10.8)	41.9 (10.4)
Female (%)	82.3	79.1^a^
Years of education (years)	12.0 (1.6)	12.3 (1.5)
Part time (%)	23.0	35.4^a^
Stickiness (%)	91.2	92.9
Gross income (€)	1.837.4 (957.2)	2133.4^a^ (872,2)
Job satisfaction	7.3 (2.0)	6.7^a^ (2.2)
Number of observations	2 430	2 684

*Notes:* the 1990 wave is used to predict turnover in 1991; standard deviations are in parentheses. Income adjusted by the Consumer price index with base 2010.

^a^Statistically different from that of nurses on at least a 5% significance level

**Fig. 2:**
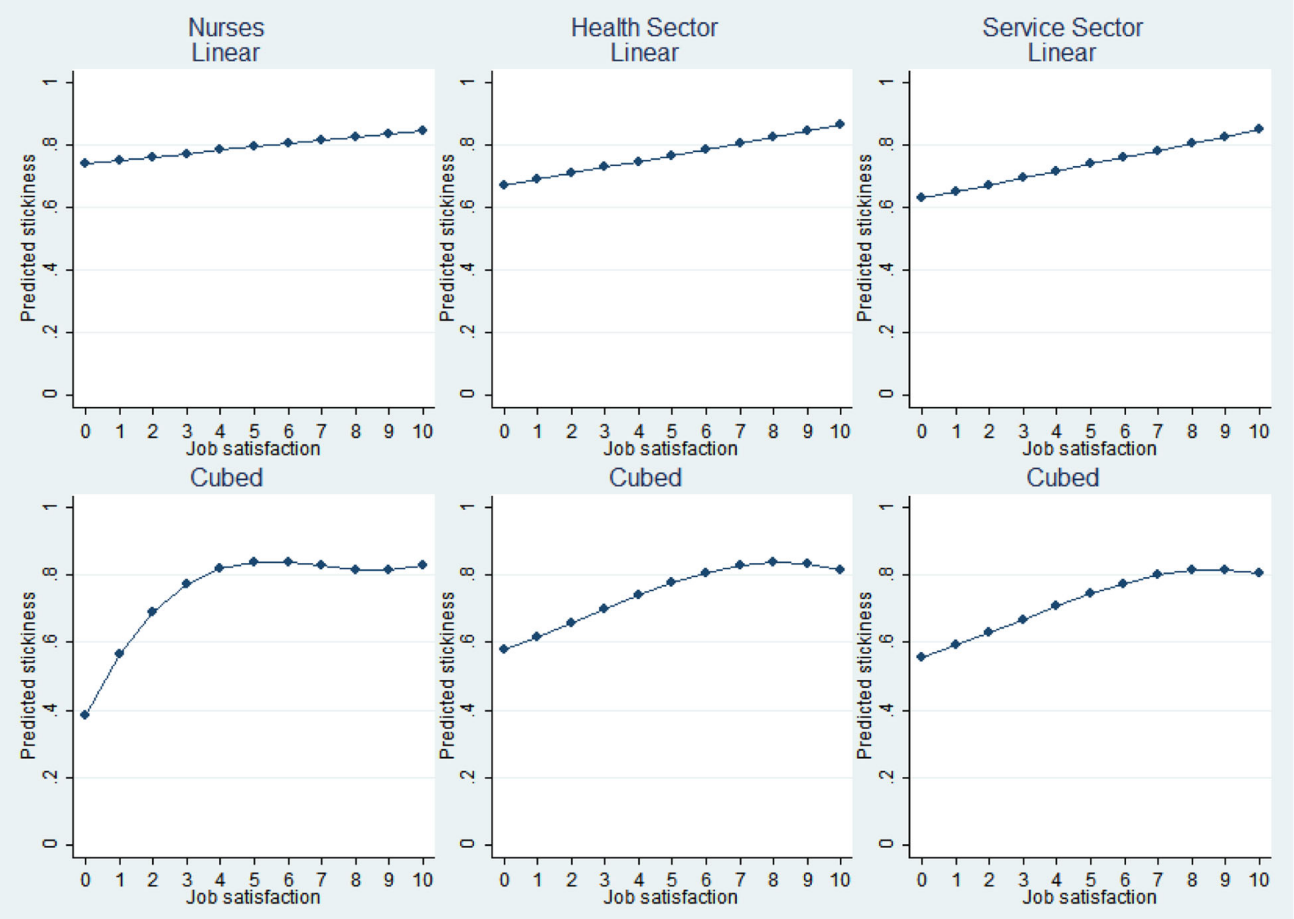
Job satisfaction and stickiness in different sectors

The declining job satisfaction simultaneous to increasing stickiness is counterintuitive given the usual association between lower job satisfaction levels and higher turnover rates. This association between job satisfaction and turnover rates is evident in Fig. 2, which plots the linear and cubed specifications for job satisfaction regressed onto job stickiness in three sectors—nursing, health, and services (with the corresponding regression results reported in Appendix 2).^1^^,^ 2 As the more flexible cubic specification shows, the relation between job satisfaction and stickiness is not linear: at a job satisfaction level of about 5 points (on a 10-point scale), the relation becomes flat. Interestingly, this leveling out in the nursing sector seems to occur at a lower job satisfaction level than in the health and services sectors, implying that retention is maintained at lower job satisfaction levels in the nursing sector.

**Fig. 3:**
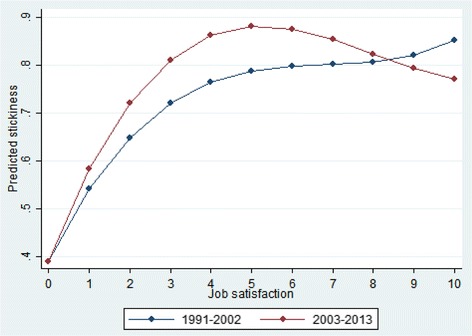
Job satisfaction and stickiness: 1991–2002 vs. 2003–2013

One possible explanation for this paradoxical observation is that the relation between job satisfaction and stickiness has changed over time. We test this conjecture by regressing job satisfaction on job stickiness separately for each of the two periods (see Fig. 3), again building in flexibility by including linear, squared, and cubic terms (see Appendix 3 for the full regression results). Although the differences are neither large nor significant at conventional levels (because of the relatively small sample sizes), the curve for the earlier period (1990–2002) lies below that for the later period (2003–2013), implying that, for a given level of job (dis)satisfaction, stickiness rates are higher in the more recent past. This observation is in line with the trends illustrated in Fig. 1.

**Fig. 4:**
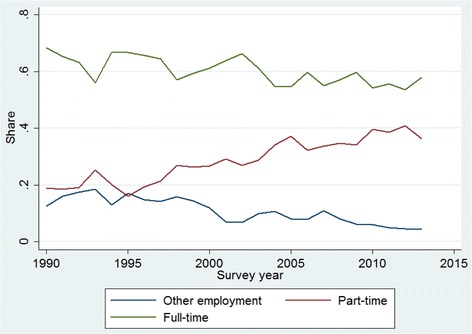
Trends for full- and part-time employment among nurses. Note: “Other employment” = nurses in training

**Fig. 5:**
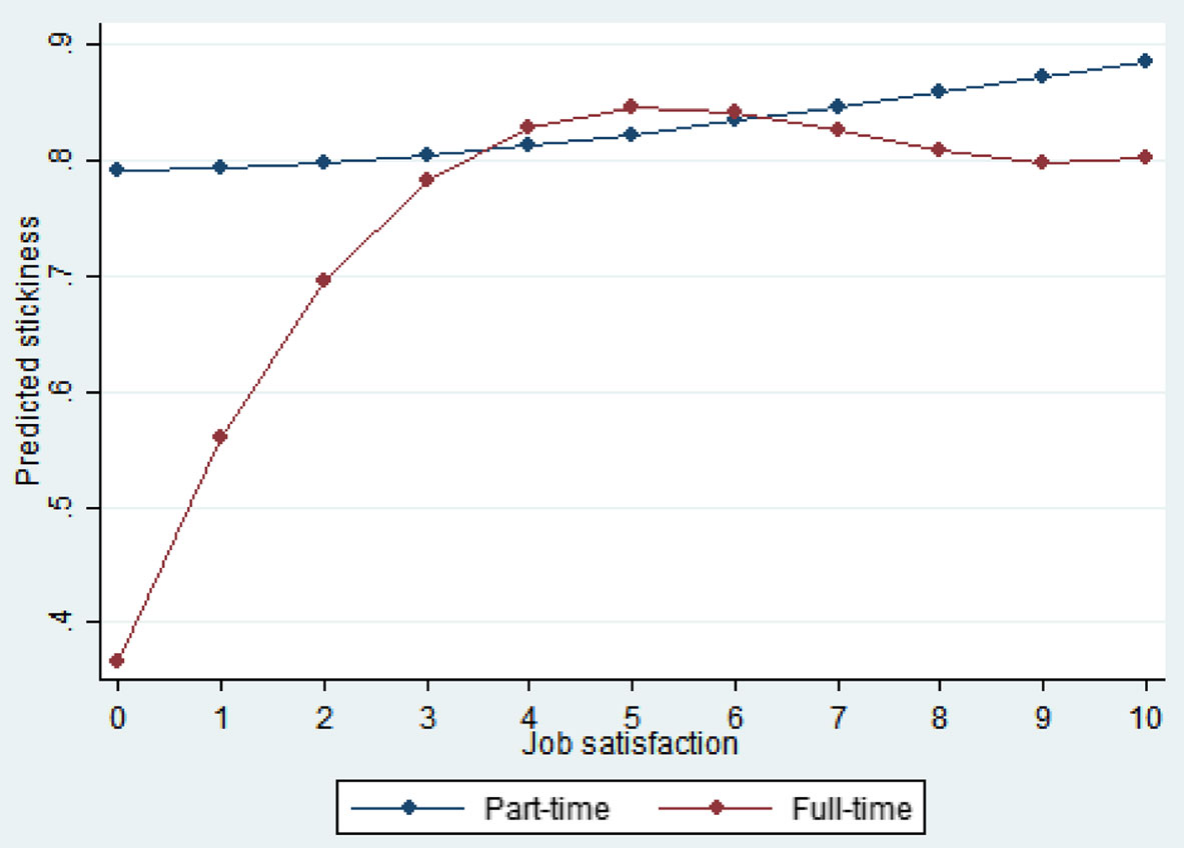
Job satisfaction and stickiness: part-time vs. full-time employment

One possible reason for this changing relation between job satisfaction and job stickiness may be the dramatic rise in part-time employment among nurses, which, as Fig. 4 shows, nearly doubled from around 20 to 40% between 1990 and 2013. Figure 5 thus depicts the relation between job satisfaction and stickiness for both part- and full-time nurses (with the full regression results given in Appendix 4). Although the relatively small sample sizes lower the analytic power, the flat relation in the case of part-time nurses supports our conjecture that their stickiness is quite resilient to changes in job satisfaction.

A final objective of this study is to assess whether the determinants of nurses’ job stickiness have changed in the past 24 years, which we do by reporting the estimated coefficients for the stickiness regressions in Table 2. As column (1) shows, in the regression for pooled data across all waves, among female nurses or those in bad health (proxied by number of doctor visits), the older the individual, the lower the job stickiness. However, with higher wages and tenure, stickiness increases. These results largely conform to the findings reported in the literature on nurse turnover. Our focus, however, is primarily on any changes in these determinants’ importance across time, so in column (3), we report the results of interacting a 2004–2013 period dummy with the main determinants of job turnover. The results suggest that both the determinants and their importance have remained stable over time. Only with regard to health is there a slight attenuating effect, implying that, given a certain number of doctor visits, stickiness is higher in the more recent past. A second significant interaction term is overtime work, which is more likely to reduce stickiness in the 2004–2013 period than in the 1991–2003 period. Nonetheless, both the magnitude and the significance levels of these interaction terms are relatively small. We thus conclude that what was important in the past still matters today.

**Table 2:** Determinants of job stickiness

	(1)	(2)	(3)
	Full specification No interaction	Joint estimation	
		Baseline 1991–2003	Interaction 2004–2013
Age	−0.002^*^	−0.003	0.002
	(0.001)	(0.002)	(0.003)
Female	−0.044^*^	−0.040	0.004
	(0.023)	(0.032)	(0.043)
Married	0.007	0.011	0.003
	(0.024)	(0.038)	(0.046)
Years of education	−0.001	−0.000	−0.005
	(0.006)	(0.009)	(0.013)
Wages/1 000	0.005^***^	0.003^**^	0.002
	(0.001)	(0.001)	(0.002)
Household income/1 000	−0.004	0.005	−0.014
	(0.009)	(0.013)	(0.017)
No. of children in household	0.031^***^	0.033^**^	−0.004
	(0.011)	(0.014)	(0.018)
No. of doctor visits	−0.002^***^	−0.003^***^	0.002^*^
	(0.001)	(0.001)	(0.001)
Years in the company	0.005^***^	0.003^*^	0.003
	(0.001)	(0.002)	(0.002)
Work hours (actual)	−0.001	−0.000	−0.002
	(0.001)	(0.002)	(0.002)
Work hours (overtime)	0.001	0.002	−0.004^*^
	(0.001)	(0.002)	(0.002)
Constant	0.982^***^	0.968^***^	−0.217
	(0.123)	(0.167)	(0.224)
State controls	Yes	Yes	
Year controls	Yes	Yes	
*N*	3 670	3 670	
adj. *R* ^2^	0.057	0.063	

*Note:* Model (1) uses pooled data from all waves and without time interaction variables. Column (2) shows the coefficients of the variables when interaction terms are included. Column (3) shows the interaction coefficients. Heteroscedastic robust standard errors clustered on the individual level are in parentheses.

*p < 0.1, **p < 0.05, ***p < 0.01

## Discussion

The findings of this study challenge some widely accepted research observations on the relation between job satisfaction and job stickiness among nurses. Most notably, analyzing stickiness and job satisfaction over approximately a quarter of a century in the German nursing market reveals that, despite falling job satisfaction levels, stickiness levels have actually increased slightly. Our analysis shows that this paradox can in part be explained by the changing relation between job satisfaction and stickiness, which in turn is related to the rise in part-time employment. We also show that the relation between job satisfaction and stickiness rates in the German context is not linear. In fact, the job satisfaction-stickiness relation levels out at relatively low levels of job satisfaction (5 on a 10-point scale), and for nurses, the leveling-out threshold is apparently lower than for other professions, implying that occupational commitment may be more pronounced in this profession.

This paradoxical relation between job satisfaction and job stickiness among nurses in the German labor market must also be viewed in the wider context of institutional changes that took place in the past decades, including nurse compensation trends, hospital downsizing trends, professional affinity by organizational type, and, as mentioned, the substantial increase in part-time employment. With respect to compensation trends, the regional differences brought about by political and structural differences between the former East and West Germany need to be examined. In 2013, for instance, the median gross wage for nurses in the East (€2931) remained lower than that in the West (€3236) [25]. In the first decade following the 1990 reunification, differences in both wages and the demand for nurses led to ongoing East-West internal mobility right up until 2003 [40]. In recent years, however, this wage gap has decreased [41], which may have reduced the incentive to change jobs and produced relatively higher stickiness among German nurses.

The resilience of stickiness despite declining job satisfaction may also be attributable to nurses’ identification with, and preference to work for, particular types of organizations, which is often dependent not only on job availability but also job fit. For example, most NGO-owned hospitals in Germany have a Christian leaning and tend to attract nurses who obtain a higher sense of intrinsic reward from serving the community [42]. This intrinsic sense of reward plus identification with a particular type of work environment may mitigate the effects of job satisfaction and lead to higher nurse stickiness despite declining job satisfaction. Other mitigating effects include the protection of seniority within an organization [43] and avoidance of having to learn a new information system to support the extensive documentation requirements expected of a German nurse [44, 45]. At the same time, the deep societal respect for the nursing profession, reflected in image ratings in recent years, may also have helped to enhance the job stickiness of nurses in the German labor market [46–53].

The increase in stickiness in the 2003–2013 period relative to the 1991–2002 period, on the other hand, may be explained by the history of active downsizing in the German hospital market. Between 1991 and 2003, in line with the early 1990’s global trend, many German hospitals were closed, downsized, or merged, resulting in an overall 8.9 and 18.6% decrease in the number of hospitals and hospital beds, respectively [40]. German nurses working in downsized hospitals either lost their jobs, decreased their work hours, or had to shift employment, which decreased their job stickiness during that period. Between 2003 and 2014, however, although hospital downsizing continued, the magnitude was relatively smaller, with only a 9.9 and 7.6% decrease in the number of hospitals and hospital beds, respectively [40].

As the above history illustrates, the job satisfaction-stickiness relation is dynamic; it changes over time in accordance with changes in the labor force structure, institutions, and policies. In particular, our study shows that part-time employees are less likely than full-time employees to leave their jobs for a given level of job satisfaction, which may explain why in Germany, which has doubled the proportion of nurses working on a part-time basis over the past two decades [40], the fall in job satisfaction has not given rise to higher fluctuation rates. It is thus plausible to hypothesize that for a given level of job (dis)satisfaction, stickiness rates are higher for part-time than for full-time nurses because dissatisfaction is more easily handled with less time spent at work and part-time nurses are often married with a husband as the main breadwinner [54]. In such households, the regional job market becomes smaller, making job changes more difficult. Of course, dissatisfied part-time nurses could also be less attached to the labor market and thus have a higher tendency to self-select out. There is, however, little evidence that (most notably) women self-select out of the labor market because of job dissatisfaction [30]. One final contributor to the growth in job stickiness among part-time employees, as well as to the increase in part-time employment itself, is the aging of the German nursing workforce since older nurses tend to show higher commitment to their employer [55].

Methodologically, our study underscores the importance of using decade-long longitudinal data to test commonly held beliefs and improve understanding of nurse turnover patterns, for it may be a “dearth of [such] studies [that] has hampered progress in the field” [56]. By using such data, we are able to show that, despite a wealth of popular anxiety and discourse on increased flexibility and mobility in labor markets, retention rates have actually risen slightly in the past two decades. This observation feeds into earlier documentation of increased job stability in Germany’s entire labor market [57, 58]. Hence, although ascertaining the optimal degree of mobility in an economy is difficult, the fact that fluctuation rates in the German nursing market have remained relatively stable in the past two decades supports the viewpoint that inefficiencies resulting from high fluctuation rates have not actually increased. In fact, some authors have argued that mobility in the German labor market may be sub-optimally low [58].

This present study is subject to certain limitations, not least of which is the detail sacrificed for the sake of using longitudinal panel data. Our data set, for example, only includes a one-item measure of job satisfaction which, although widely employed, may slightly impair validity. We also have limited information on such factors as job characteristics and employment motivation, which would also be interesting to analyze longitudinally. Finally, although our entire sample is reasonably large (approximately 1000 nurses), our annual samples are quite small (see Appendix 1), which negatively affects the power of certain analyses. Thus, some estimated coefficients are not significant at conventional levels, which warrant consideration when interpreting the results.

## Conclusions

Nevertheless, the fact that the GSOEP is one of the longest running representative panel surveys in the world permits a more extended assessment of nurse job satisfaction trends than achieved by any other study we know. Such an assessment is essential to better understand the role of health policies and to inform future policy decisions that support the job satisfaction and efficiency of the German nursing workforce.

The paradoxical relation between job satisfaction and job stickiness in the German nursing context could be explained by historical downsizing trends in hospitals, an East-West German nurse compensation gap, and an increase in the proportion of nurses employed on a part-time basis. A clearer analysis of each of these trends is thus essential for the development of evidence-based policies that enhance the job satisfaction and efficiency of the German nursing workforce.

## Appendix 1

**Table 3:** Sample size per wave

Year	1990	1991	1992	1993	1994	1995	1996	1997
#nurses	126	134	140	133	138	180	164	177
Year	1998	1999	2000	2001	2002	2003	2004	2005
#nurses	198	173	312	270	285	253	283	252
Year	2006	2007	2008	2009	2010	2011	2012	2013
#nurses	264	248	232	240	211	227	223	251

## Appendix 2

**Table 4:** Job satisfaction and stickiness in different sectors: OLS regressions

	Nursing		Health		Services	
	(1)	(2)	(3)	(4)	(5)	(6)
Job satisfaction	0.011**	0.211***	0.019***	0.037	0.022***	0.032
	(0.005)	(0.050)	(0.004)	(0.043)	(0.002)	(0.022)
Job satisfaction^2^		−0.031***		−0.003		−0.003
		(0.009)		(0.007)		(0.004)
Job satisfaction^3^		0.002***		−0.0004		−0.0004
		(0.001)		(0.0004)		(0.0002)
*N*	4 215	4 215	11 091	10 191	66 791	66 791
Adjusted *R* ^2^	0.036	0.045	0.018	0.022	0.023	0.025

*Note:* The regression includes state and year dummies as additional controls. Heteroscedastic robust standard errors clustered on the individual level are in parentheses. * *p* < 0.1, ** *p* < 0.05, *** *p* < 0.01

## Appendix 3

**Table 5:** Job satisfaction and stickiness 1990–2002 vs. 2003–2013: OLS regressions

	1990–2002 baseline	2003–2013 interaction
Job satisfaction	0.174***	0.049
	(0.062)	(0.105)
Job satisfaction^2^	−0.025**	−0.007
	(0.012)	(0.019)
Job satisfaction^3^	0.001*	0.001
	(0.001)	(0.001)
*N*	4 215	
Adjusted *R* ^2^	0.040	

*Note:* The regression includes state and year dummies as additional controls. Heteroscedastic robust standard errors clustered on the individual level are in parentheses. **p* < 0.1, ***p* < 0.05, ****p* < 0.01

## Appendix 4

**Table 6:** Job satisfaction and stickiness part-time vs. full-time—OLS regressions

	Part-time baseline	Full-time interaction
Job satisfaction	0.001	0.224*
	(−0.118)	(−0.133)
Job satisfaction^2^	0.001	−0.035
	(−0.020)	(−0.023)
Job satisfaction^3^	0.000	0.002
	(−0.001)	(−0.001)
Constant	0.561**	−0.423*
	(−0.230)	(−0.257)
*N*	3 788	
Adjusted *R* ^2^	0.046	

*Note:* The regression includes state and year dummies as additional controls. Heteroscedastic robust standard errors clustered on the individual level are in parentheses. **p* < 0.1, ***p* < 0.05, ****p* < 0.01

## Abbreviations

FTE: Full-time equivalent
GSOEP: German Socio-Economic Panel

## Footnotes

1 Documentation on data quality is available at https://www.diw.de/en/diw_02.c.222842.en/data_quality.html.

2 The healthcare sector includes other health related occupations such as doctors and therapists, based the classification of the German Census Office (StaBuA 1992 code 8400-8600). The total service sector sample excludes healthcare and nurses.
